# Psychical research and the origins of American psychology: Hugo Münsterberg, William James and Eusapia Palladino

**DOI:** 10.1177/0952695112439376

**Published:** 2012-04

**Authors:** Andreas Sommer

**Affiliations:** University College London, UK

**Keywords:** boundary-work, discipline formation, fraud, historiography, popularization of science

## Abstract

Largely unacknowledged by historians of the human sciences, late-19th-century psychical researchers were actively involved in the making of fledgling academic psychology. Moreover, with few exceptions historians have failed to discuss the wider implications of the fact that the founder of academic psychology in America, William James, considered himself a psychical researcher and sought to integrate the scientific study of mediumship, telepathy and other controversial topics into the nascent discipline. Analysing the celebrated exposure of the medium Eusapia Palladino by German-born Harvard psychologist Hugo Münsterberg as a representative example, this article discusses strategies employed by psychologists in the United States to expel psychical research from the agenda of scientific psychology. It is argued that the traditional historiography of psychical research, dominated by accounts deeply averse to its very subject matter, has been part of an ongoing form of ‘boundary-work’ to bolster the scientific status of psychology.

Eeny, meeny, miny mo,
Catch Eusapia by the toe
If she hollers, then we know,
James’s doctrines are not So!^1^

## Introduction: psychical research and the ‘new psychology’

At the end of the 19th century, psychical researchers such as Frederic and Arthur Myers, Edmund Gurney, Julian Ochorowicz, Charles Richet, Max Dessoir, Albert von Schrenck-Notzing, Richard Hodgson and Henry and Eleanor Sidgwick were actively involved in the making of the fledgling science of psychology. Psychical researchers initiated and organized the International Congresses of Physiological/Experimental Psychology (Alvarado, forthcoming; Nicolas and Söderlund, 2005; Plas, 2000), and they devised methodological innovations such as randomized study designs (Hacking, 1988). They contributed important empirical findings by conducting the first experiments investigating the psychology of eyewitness testimony (Hodgson and Davey, 1887), empirical and conceptual studies illuminating mechanisms of dissociation and hypnotism (Alvarado, 2002; Ellenberger, 1970; Gauld, 1992; Shamdasani, 1993) and experiments and large-scale surveys undermining the notion of dissociation and hallucinations as intrinsically pathological phenomena (Sommer, 2011; Williams, 1985). While rooted in attempts to test controversial claims of telepathy, clairvoyance and survival of death, these contributions enriched early psychological knowledge quite independently of the still hotly debated evidence for ‘supernormal’ phenomena.

Nothing epitomizes the ambivalent relationship of academic psychology to psychical research clearer than two figures generally considered as the very founders of modern psychology, William James and Wilhelm Wundt. Whereas Wundt had publicly and programmatically rejected psychical research as intrinsically unscientific in the same year he established German experimental psychology in Leipzig (Wundt, 1879), James sought to integrate it into nascent American psychology. James made original contributions to psychical research and regularly collaborated and corresponded with British and French psychical researchers (Skrupskelis and Berkeley, 1992–2004; James, 1986). In 1884, he became a founding member of the American Society for Psychical Research (ASPR) and, in 1894 and 1895, a president of the British Society for Psychical Research (SPR), and he reviewed and defended the work of the SPR in psychology and science periodicals like *Mind*, the *Psychological Review*, *Nature* and *Science*.^2^

In the United States, several of Wundt’s students, such as Hugo Münsterberg, Stanley G. Hall, Edward Titchener and James McKeen Cattell (along with other leading US psychologists not trained by Wundt), ruthlessly combated the father of American psychology in his attempts to integrate psychical research into nascent psychology (Bjork, 1983; Bordogna, 2008; Coon, 1992; Taylor, 1996). Divided by epistemological, methodological and political disagreements as well as by personal animosities (see, for example, Sokal, 1992; Taylor, 1994), leading US psychologists found themselves in rare unison agreeing that psychical research was not to be associated with the ‘new psychology’. Hence, the aggressive rejection of psychical research as the ‘unscientific Other’ of academic psychology, which James’ opponents perceived as a threat to rationality and the scientific and social order, was a vital unifying principle aiding early psychologists to achieve something like a scientific identity (Leary, 1987). 

Joseph Jastrow, one of the most active popularizers of the ‘new psychology’ in America, identified vital boundary issues of psychology when reminiscing about the problem of psychical research, ‘which in the closing decades of the nineteenth century was so prominent that in many circles a psychologist meant a “spook hunter”’ (Jastrow, *Autobiography*, in Carl Murchison [ed.] *A History of Psychology in Autobiography*, vol. 1 [Worcester, MA: Clark University Press, 1930], pp. 135–62, cited in Moore, 1977: 166–7). One can thus easily imagine how James must have embarrassed many colleagues by stating, for example, in his *Science* review of an early SPR study of telepathic hallucinations that the scholarship displayed therein comprised a combination of outstanding intellectual virtues ‘not found in every bit of so-called scientific research that is published in our day’ (James, 1887: 18). ‘Enlightened’ psychologists were also hardly amused by the founder of American psychology exclaiming in the *Psychological Review* that ‘the concrete evidence for most of the “psychic” phenomena under discussion is good enough to hang a man twenty times over’ (James, 1896: 650). 

Among those who felt driven to protest against James’ lack of epistemological squeamishness was James McKeen Cattell. As the editor of *Science*, Cattell concluded a series of heated discussions with James about a recent SPR report on the medium Leonora Piper in the pages of his journal by stating that he had attacked James

… only because I believe that the Society for Psychical Research is doing much to injure psychology. The authority of Professor James is such that he involves other students of psychology in his opinions unless they protest. We all acknowledge his leadership, but we cannot follow him into the quagmires. (Cattell, 1898: 642)^3^

It is on the backdrop of these boundary disputes that certain historical episodes which have been celebrated as victories of American scientific psychology over psychical research deserve a reassessment. Among the most widely promulgated success stories of psychology expelling its unloved sibling from academia were the public ‘exposures’ by two leading US psychologists, Hugo Münsterberg and G. Stanley Hall, of two subjects most extensively investigated by psychical researchers of the time: the Italian ‘physical medium’ Eusapia Palladino in 1909 and the American ‘mental medium’ Leonora Piper in 1910. While the Hall–Piper episode will be reserved for a separate study, this article analyses Münsterberg’s celebrated exposure of Eusapia Palladino. 

## Hugo Münsterberg, William James and Eusapia Palladino

### Palladino and James

Whereas traditional standard accounts of psychical research have portrayed proponents of the controversial discipline as gullible victims of a desperate will to believe or as otherwise intellectually or morally impaired (e.g. Alcock, 1981; Hall, 1962), less ideologically committed historical research has revealed a wide range of epistemic and metaphysical positions within the controversial discipline (Gauld, 1968; Mauskopf and McVaugh, 1980; Noakes, 2005; Oppenheim, 1985; Williams, 1984; Wolffram, 2009). Rather than favouring superficial mono-causal attributions, by, for example, explaining interest in psychical research in terms of a ‘flight from reason’ and irrational obsession with the ‘occult’, these studies have shown that scholars had not only differing motivations leading to their involvement in psychical research, but – not unlike early academic psychologists – they were also divided by competing research programmes and epistemic presuppositions.^4^

Crude axes around which to allocate research activities within the psychical research community were, for example, the question of post-mortem survival versus telepathy and clairvoyance among the living, and the study of physical versus mental phenomena. Focal points of research differed nationally as well. For instance, while the British SPR – especially under the leadership of Henry Sidgwick – favoured the study of mental rather than physical phenomena, French and Italian researchers like Charles Richet and Cesare Lombroso investigated both areas. As Heather Wolffram (2009) has shown, early-20th-century German psychical research was heavily dominated by studies in physical mediumship through the influence of the wealthy physician and former pioneer of hypnotism and sexology, Albert von Schrenck-Notzing. While the automatic speaking and writing of the American trance medium Leonora Piper – discovered and introduced to the psychical research community by William James in the late 1880s – became the most thoroughly studied mental mediumistic phenomena of all time, the Neapolitan Eusapia Palladino (1854–1918, Figure 1) was the undisputed queen of physical mediumship, puzzling some of the leading scientists and philosophers of her time.^5^

**Figure 1.**
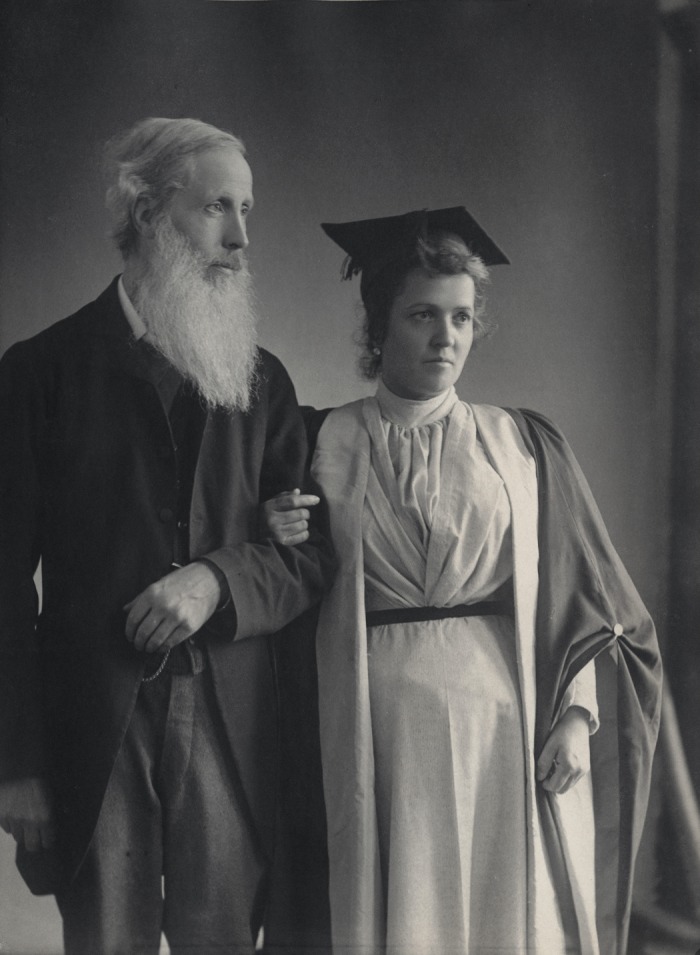
Eusapia Palladino and Henry Sidgwick in Cambridge in 1895 (Henry Sidgwick; Eusapia Palladino by Eveleen Myers (née Tennant), platinum print, circa 1890 © National Portrait Gallery, London)

According to her main investigators, such as the Polish philosopher-psychologist Julian Ochorowicz, the French physiologist Charles Richet, the British physicist Oliver Lodge and the Italian psychiatrist Enrico Morselli, Palladino’s performances were a strange mixture of blatant fraud and genuine ‘supernormal’ phenomena. While Eusapia^6^ would cheat shamelessly whenever she got the opportunity, she was also reported to have produced, sometimes in bright light and under good conditions of observation and experimental control, levitations and remote manipulations of objects, materializations of human forms and the development of bizarre pseudopodia. Many sceptical scientists who came to investigate her left as believers. For example, Cesare Lombroso, one of the arch-enemies of psychical research and spiritualism in Italy, attended sittings with Palladino in the 1890s to expose her tricks, but left completely converted (Lombroso, 1909). While Lombroso not only came to believe in the reality of Eusapia’s phenomena but also embraced the spirit hypothesis to explain some of them, most other investigators of Palladino and other mediums, such as Charles Richet, Enrico Morselli, Théodore Flournoy and Albert von Schrenck-Notzing, rejected the spirit hypothesis and favoured a psychodynamic explanation in terms of ‘teleplasty’ or ‘ideoplasty’, describing the grotesque phenomena as ‘externalized dreams’ of physical mediums. 

Not exactly conforming to the Victorian stereotype of the etheric spiritual medium, Palladino, an uneducated peasant woman, was notorious for her erratic and vulgar behaviour inside and outside her seances and trance states. For example, apart from displaying a diva-like behaviour, she would, apparently merely to entertain herself, tell obvious lies and openly flirt with some of her distinguished male investigators, sometimes jumping on the horrified savants’ laps. In a comprehensive treatise on the psychology and physical phenomena of Palladino, Enrico Morselli (1908) thus testified both to her supernormal abilities and to her hysteria, a verdict shared by other researchers who had openly reported instances of fraud in Eusapia and other physical mediums while claiming a robust residue of genuine phenomena. Rather than reducing the phenomena of physical mediumship to fraud, these investigators tried to distinguish between deliberate malicious fraud, quasi-pathological trickery outside the trance state, unconscious fraud in the trance state (where ‘dissociated streams’ of a medium’s unconscious were believed to act out auto-hypnotic suggestions to produce phenomena no matter how) and the alleged supernormal phenomena not thus explicable. These researchers viewed mediumistic fraud of a certain order not only as relatively easy to control in an experimental setting, but also as a field of psychological study in its own right.^7^

Though he never conducted formal experiments with Eusapia, James had been following the reports by his colleagues in Europe.^8^ Commenting on constant alternations of news regarding Palladino’s exposures on the one hand and confirmations of the reality of her phenomena on the other, on 30 April 1903 James wrote to his friend and colleague Théodore Flournoy: ‘Forever *baffling* is all this subject, and I confess that I begin to lose my interest’ (Skrupskelis and Berkeley, 1992–2004: X, 239).^9^ Four years later, after studying confirmatory reports by French researchers, James wrote to Eleanor Sidgwick that now to him ‘the proof seems overwhelming, and it has been an enormous relief to my mind to quit the balancing attitude which I have voluntarily maintained for 15 years, and come to a stable belief in the matter’ (1 August 1907, Skrupskelis and Berkeley, 1992–2004: XI, 405–6). 

Among the studies in support of the reality of some of Eusapia’s phenomena has been the report by the Britons Everard Feilding, William Baggally and Hereward Carrington (1909). Initially sceptical, the experimenters, expert conjurors and enjoying a reputation as debunkers of fraudulent mediums, came to Naples to expose Palladino, but after a series of 11 experimental sittings concluded that among the usual obvious trickery there was a range of apparently genuine phenomena defying explanation. After Naples, Carrington, a freelance researcher and science journalist with little if any regular income, arranged for Palladino to travel to the USA and be investigated by committees of scientists to bolster the scientific status of psychical research and, although he repeatedly denied this, probably also to secure some financial gain for himself.^10^ He invited the leading papers to report the results, and the American Palladino experiments were among the major stories in the *New York Times* in late 1909 to early 1910. 

However, the project turned out to be a fiasco for Palladino, Carrington and psychical research at large. As usual, Palladino behaved erratically and cheated blatantly. She also upset her sponsors, e.g. by suddenly cancelling a feverishly anticipated seance in the *Times* tower in New York. Despite occasional positive coverage in the press, the heaviest blow Palladino received in America was a report by the German-born Harvard psychologist Hugo Münsterberg, claiming to have exposed the great medium once and for all.^11^ The report, originally published in the *Metropolitan Magazine* (Münsterberg, 1910, reprinted with minor changes in Münsterberg’s *American* *Problems*, 1912), was summarized in the *New York Times* and many other papers across and beyond the country and promulgated in both the popular and scientific press as the final word on physical mediumship in general and Eusapia in particular. 

### Hugo Münsterberg and psychical research

Münsterberg was one of several students of Wilhelm Wundt who were to become pillars of American psychology.^12^ In 1893, responding to competition from other universities, William James persuaded the gifted experimentalist to come to America and run the laboratory of experimental psychology at Harvard. In Germany, like Wundt, Wilhelm Preyer and other early German psychologists, Münsterberg had recognized the importance of popularizing the nascent science by publicly demarcating the ‘new psychology’ from psychical research. Still in Freiburg, Münsterberg had given a popular lecture, ‘*Gedankenübertragung*’ [Thought-transference], which was published in the *Berichte der Naturforschenden Gesellschaft zu Freiburg* and subsequently as a pamphlet. He attacked the work of the psychological societies in Munich and Berlin in psychical research, which, he warned, fatally reinforced the popular view of the identity of psychology and spiritualism and other superstitions posing serious threats to modern science and civilization.^13^ Without naming them, he also scolded certain eminent scientists who had stated that psychic phenomena were not yet confirmed sufficiently, whereas, he complained, it would have been their scientific duty to state ‘in plain language: they are impossible!’ (Münsterberg, 1889: 3; my translation). As in later writings on psychical research, Münsterberg tried to stress the essential difference between scientific psychology and psychical research on the one hand, and between psychical research, which he constantly conflated with vulgar spiritualistic belief systems, and true religion on the other.^14^

Ten years after his Freiburg talk, Münsterberg, now in charge of experimental psychology at Harvard, published the essay ‘Psychology and Mysticism’ in the popular *Atlantic Monthly*. Essentially repeating the basic themes of ‘*Gedankenübertragung*’, the gist of the article was that science had explained all reported supernormal phenomena in terms of hypnotism, hysteria, muscle reading, hyperaesthesia, dissociation, hallucinations and illusions ‘and other mental states which psychology understands just as well as it does the normal associations and feelings’ (Münsterberg, 1899: 75), neglecting to mention that it was psychical researchers rather than experimental psychologists who had made major contributions to these areas. Münsterberg admitted that he had ‘never taken part in a telepathic experiment or in a spiritualistic séance’, justifying his reluctance to gain first-hand experience by referring to ‘experiences of some friends’, who ‘had spent much energy and time and money on such mysteries, and had come to the conviction that all was humbug’ (ibid.: 77). Münsterberg claimed that the only time he wavered was when he had

… received a telegram from two telepathists [*sic*] in Europe, asking me to come immediately to a small town where they had discovered a medium of extraordinary powers. It required fifteen hours’ traveling, and I hesitated; but the report was so inspiring that I finally packed my trunks. Just then came a second message with the laconic words, ‘All fraud’. Since that time I do not take the trouble to pack. I wait quietly for the second message. (ibid.: 77)

James was hardly impressed by the *ex cathedra* pronouncements of his colleague, which, in a letter to Harry N. Gardiner on 19 January 1899, he described as strategically ‘clever’ but ‘essentially childish’ (Skrupskelis and Berkeley, 1992–2004: VIII, 484). Viewing Münsterberg’s article as yet another example of rhetorical trickery he had decried previously in colleagues like Hall, Titchener, Cattell and Jastrow, James wrote: ‘The insolence of these fellows, sure of the applause of Scientism, whatever they may say, is amusing’ (ibid.). Moreover, regarding Münsterberg’s self-professed eagerness to investigate alleged supernormal phenomena, James revealed to Edward Titchener on 21 May 1899:

My colleagues for the most part, when invited, have simply refused to see Mrs. Piper [whom James had hosted to conduct a series of experiments]. [Josiah] Royce, e.g., who had only to step from the next door but one into my house. Munsterberg said it was no use; if he got such results, he would know himself to have been hypnotized. I said ‘bring your wife, sit in the corner & observe, and see if your accounts agree’. He replied ‘I should never allow my wife to visit such a performance’. I call that real sportsmanlike keenness for new facts! (Skrupskelis and Berkeley, 1992–2004: VIII, 532)^15^

For whatever reasons, Münsterberg apparently changed his mind at the end of the same year and asked for sittings with Mrs Piper. In a letter to James’ fellow pragmatist, the German-born Oxford philosopher Ferdinand Canning Schiller, James wrote on 11 October 1899: ‘He certainly ought not to be allowed to see Mrs. Piper. He will be hypnotized, if he gets anything – if not, he will have exploded the phenomenon. It is too late!’ (Skrupskelis and Berkeley, 1992–2004: IX, 59).

In England, Schiller had published a satirical analysis of Münsterberg’s article in the SPR *Proceedings* (Schiller, 1899), as well as a rather vitriolic review (ridiculing the bad English) of his compatriot’s *Psychology and Life* in the journal *Mind*. Münsterberg complained to James about Schiller and asked for support. In his reply on 17 November 1899, James agreed that Schiller’s ridicule of Münsterberg’s English was below the belt and reassured him that he had scolded Schiller accordingly.^16^ Regarding Schiller’s review of Münsterberg’s mysticism essay, however, James wrote to Münsterberg that he had

… no just cause of complaint. Your mysticism article, so to speak with perfect candour, seems to me a monumentally foolish performance. The time is passed for metaphysical dogmatism about natural phenomena and I think it was a great compliment that he should have discussed your paper at all. If discussed, how could it be discussed but in a comic vein? Pardon these sentiments, my dear colleague; you can easily understand them; brevity forces me to be blunt. (Skrupskelis and Berkeley, 1992–2004: IX. 86)^17^

A decade later, on 15 November 1909, the *New York Times *headlined that Münsterberg had accepted Hereward Carrington’s invitation to serve on a scientific committee investigating the phenomena of Eusapia Palladino. Münsterberg was quoted thus: ‘I will willingly serve with a committee of scientists to determine the limitations of the medium Paladino’. This was because he was ‘intensely interested … in psychologic phenomena of all descriptions, and if this woman is all she is accounted, I think I as well as my fellow-scientists will know our time well spent in watching her powers’.^18^

Carrington had also invited James to participate in the media spectacle. In his reply on 15 June 1909, James declined the invitation:

My small remaining energy has to go elsewhere. You’ll think me a mollycoddle (or whatever the translation of that Rooseveltian term into this sphere of life may be) but I have a constitutional antipathy to the newspaper manner of *explaining* all such things, and believe that they had better make their way gradually into more scientific circles first, and from thence later down. Eusapia has had the good luck so far to follow that line of success. After reading Courtier’s report, it seemed to me that it was quite unnecessary for duffers like myself to *see* E. P. at all. They had done more than I could ever possibly do to verify.^19^

James then issued a warning regarding Carrington’s invitation to Münsterberg, Hall, Jastrow and other psychologists hostile to psychical research to investigate Palladino. He stated that if they ‘would investigate seriously, it would be a fine thing for you to get her here for them. But I have very little faith in the candor of such men’, doubting any useful results forthcoming from such a cooperation. Based on his previous experiences, James concluded his letter by stating that he did not ‘wish to take any trouble to convince such men as Münsterberg and Jastrow’, and, uncharacteristically harsh, he advised Carrington: ‘Let them perish in their ignorance and conceit.’^20^

### Münsterberg’s ‘exposure’

Aware of his immense popularity and visibility in the American press, Münsterberg must have known that his verdict on any controversial matter related to the study of the human mind would be snapped up by the media and accepted as the official verdict of scientific psychology.^21^ Hence, like Münsterberg’s previous writings on psychical research, his Palladino article, written after attending two sittings on 13 and 18 December 1909, revealed his determination to cleanse academic psychology from any occult connotations at all costs and to promote psychology as an applied and thus useful science at the same time. Commenting on his previous refusals to actively investigate psychic claimants, Münsterberg wrote that, daily ‘urgent requests’ notwithstanding, he had ‘remained loyal to my program and refused consistently all contact with the mystical phenomena’ (Münsterberg, 1912: 119). Explaining his sudden change of mind, wrote: It is the duty of a psychologist to examine the totality of mental occurrences, and he has no right to close his eyes on that which seems to transcend our present powers of explanation. I heard this so often and so impressively that I finally yielded. I simply said: ‘Madame Palladino is your best case. She is the one woman who has convinced some world-famous men. I never was afraid of ghosts; let them come!’ (ibid.: 120).

Münsterberg claimed that his scientific training, which, he stressed, entirely rested on trust, would render him incapable of discovering Palladino’s cunning tricks, an explanation he offered for the conversion of other scientists who had declared Palladino’s phenomena real. Again blurring the distinction between empirical research and ideological spiritualism, and neglecting to acknowledge experimental conditions in previous series of experiments reported by non-spiritualists such as Morselli, Richet, Flournoy and the Curies, Münsterberg characterized the Palladino sittings uniformly:

Always the same silly, freakish, senseless pranks repeated on thousands of nights before small groups of more or less superstitious people under conditions of her own arrangement, conditions entirely different from ordinary life, with poor illumination and with complete freedom to do just what she pleases. (ibid.: 135–6)

Later the reader finds an admission regarding investigators’ attempts to automatize control and thus obtain results independent of the pitfalls of human observation, contradicting Münsterberg’s previous statement. But Münsterberg had ‘no sympathy with the efforts to raise the level of the investigation by introducing subtle physical instruments. That gives to the manifestations an undeserved dignity and withdraws the attention from the center of the field’ (ibid.: 140). After speculating about how Palladino might fake her phenomena, Münsterberg wrote:

Of course, there will be some who in reply will fall back on their old outcry that all this is dogmatism and that instead of mere theories of explanations they want actual proof. I am afraid I must be still clearer there. I must report what happened at the last meeting which I attended. (ibid.: 141)

Münsterberg then related how he and other sitters controlled Palladino’s hands and feet,

… and yet the table three feet behind her began to scratch the floor and we expected it to be lifted. But instead, there suddenly came a wild, yelling scream. It was such a scream as I have never heard before in my life, not even in Sarah Bernhardt’s most thrilling scenes. (ibid.: 142)^22^

Münsterberg then breaks the ‘suspense’:

What happened? Neither the medium nor Mr. Carrington had the slightest idea that a man was lying flat on the floor and had succeeded in slipping noiselessly like a snail below the curtain into the cabinet. I had told him that I expected wires stretched out from her body and he looked out for them. What a surprise when he saw that she had simply freed her foot from her shoe and with an athletic backward movement of the leg was reaching out and fishing with her toes for the guitar and the table in the cabinet! (ibid.: 143)

Münsterberg’s conclusion, to be readily promulgated in academic and popular channels of information alike, was therefore: ‘Her greatest wonders are absolutely nothing but fraud and humbug; this is no longer a theory but a proven fact’ (ibid.: 144). 

At the same time, however, he proposed that Eusapia might not be held fully responsible for her cheating. For Münsterberg explained that it was ‘improbable that Madame Palladino, in her normal state is fully conscious of this fraud. I rather suppose it to be a case of complex hysteria in which a splitting of the personality has set in’ (ibid.: 144). Hence, what previous researchers of physical mediumship had long come to view as a psychological problem that deserved to be studied in its own right, Münsterberg falsely claimed as his original contribution to psychology: the discovery of ‘unconscious’ mediumistic trickery. Rather than viewing ‘unconscious fraud' as a confounding but controllable variable, however, Münsterberg proposed it as a sufficient explanation for the whole complex of phenomena studied by investigators of physical mediumship.

On 22 January 1910, William James sent a copy of Münsterberg’s article to Oliver Lodge in England, commenting on

… the depth to which the ‘scientific’ mind can descend, in the person of my impudent colleague Munsterberg. It is a buffoon article, as if written by a bagman. The worst of it is that I can imagine no process by which he could possibly be made ashamed of it. So essentially dogmatic is his mind that he will remain convinced to the end that he has ‘exposed’ Eusapia and be proud of the literary performance. Absolutely the *only* ‘observation’ was the catching of the foot by the man on the floor. M——g insinuates that this was done in consequence of his advice, but in point of fact he knew nothing about it till he was told after the sitting. (Skrupskelis and Berkeley, 1992–2004: XII, 418)

On 26 January 1910, James wrote to Théodore Flournoy:

There is no limit to his genius for self-advertisement and superficiality. Mendacity too! He would have the readers think that Morselli, Bottazzi, Ochorowicz, Richet et al are ‘spiritualists’, and by lugging in pragmatism (!) he tries to insinuate that I am also one. (ibid.: 423)^23^

In another letter to Flournoy on 9 April 1910, James reinstated the previous allegations regarding Münsterberg’s claims to responsibility for Palladino’s exposure:

The gentleman who seized her foot was a stranger to M——g, and none of the company knew what had happened till after the sitting was over, when he informed M——g and one or two others. M——g tells everybody (or gives them to believe) that this man was his employé, acting by his direction! In point of fact he was one of the guests whose payment made it possible for Carrington to invite M——g *gratis.* (ibid.: 466)^24^

Prior to the publication of Münsterberg's *Metropolitan Magazine* article, rumours of the impending publication had reached Hereward Carrington, who wrote to Münsterberg on 6 January 1910, asking him to withhold publication before a fuller record of the series was published:

… your remarks at the time, as shown in the stenographic notes, and your subsequent utterances to Mrs. Carrington, myself and other sitters at the conclusion of the séance, indicated clearly enough that you believed and, in fact, stated at the time that the case was of great interest, scientifically, and that the phenomena were, in a large part, at least, not due to fraud on the part of the medium. If your opinions have since changed, this must be due to some cause or causes which I think you should state.^25^

Years later, Carrington concluded his final published analysis of the Münsterberg–Palladino affair thus: ‘Inasmuch as I wrote a letter to Professor Münsterberg, at the time, accusing him of willful falsehood, I can see no reason to refrain from repeating that assertion here. His own dictated statement to the stenographer refutes his claim’ (Carrington, 1957: 246).^26^

In fact, if we were to believe the published minutes of the sittings, there were more problems with Münsterberg’s ‘exposure’ than those identified by James. For example, contrary to what Münsterberg’s article implied, nobody except Eusapia herself had claimed that her foot was grabbed, and all sitters at the time denied such action (Carrington, 1954: 113, 117). Furthermore, while Münsterberg implied that Eusapia’s scream marked the cessation of the sitting on 18 December, the experiment not only continued uninterrupted for another 17 minutes (ibid.: 114), he also neglected to mention that the cry following the alleged foot-grabbing incident at 11.44 (‘E. screams sharply. Reason not known’, ibid.: 113) was not the first one. According to the minutes, at 11.01 Eusapia had cried ‘as if in pain’ and wept ‘as if physically hurt’ (ibid.: 111).^27^ The minutes also state that at the time of the alleged exposure, Münsterberg, who controlled Palladino’s hands, and Professor Bumpus (a friend of Münsterberg), controlling both of her feet, explicitly stated: ‘control is all right’ (ibid.: 113).

Physical mediumship in general, and the Palladino case in particular, posed a complex enough problem to trained scientists and clinicians open to the possibility of the genuineness of Eusapia’s feats. Obviously, mediums and their investigators were particularly easy prey for determined debunkers such as Münsterberg, Jastrow and others, who could be sure that the popular and academic press alike would accept their verdicts as scientifically authoritative. Hence, James, who commented on Palladino’s behaviour in America to Flournoy that ‘Eusapia’s type of performance is *detestable* – if it be not fraud simulating reality, it is reality simulating fraud!’ (9 April 1910, Skrupskelis and Berkeley, 1992–2004: XII, 466) was not the only psychical researcher who had doubts regarding the value of Carrington’s project. The philosopher and psychical researcher James Hyslop exemplified disagreements in the psychical research community over the scientific merit of physical mediumship in general and Palladino in particular when he wrote:

The Palladino case, as it has been managed, is not calculated to influence intelligent people who have no time to spend years and fortunes on it. It only excites dispute and many of the facts asserted of it are so closely related to fraud that even the apology of hysteria has little effect. (Hyslop, 1910: 182–3)^28^

After James’ death, Münsterberg continued his crusade to expel psychic phenomena from the epistemological and professional territories of psychology. His last debunking exercise in the name of scientific psychology was another *Metropolitan Magazine* article in 1913, an enlarged version of which he incorporated in *Psychology and Social Sanity* (Münsterberg, 1914), which outlines his investigations of Beulah Miller, a 10-year-old girl in Warren, Rhode Island. Newspaper reports had claimed that the girl was able to read minds and perceive remote or hidden objects. After rejoicing that ‘organizations for antilogical, psychical research eke out a pitiable existence nowadays’, Münsterberg related that he had undertaken his investigation of Beulah Miller from the same ‘feeling of social responsibility’ in which he had ‘approached the hysterical trickster, Madame Palladino, who had so much inflamed the mystical imagination of the country’ (ibid.: 143). Münsterberg proposed that Bleulah's feats were to be explained by a pathological hypersensitivity, which enabled her to unconsciously perceive and decode subtle sensory clues by members of her family and other persons in possession of the information Beulah was to present. Though not a fraud, Münsterberg explained, the girl’s ‘mental makeup in this respect constantly reminds the psychologist of the traits of a hysteric woman’ (ibid.: 172). 

## Conclusion: historiography as boundary-work

Pointing, for a change, the spotlight that orthodox critics have put on scientific deviants back on the critics themselves, a large can of worms threatens to explode in the historian’s face. For not only does the Münsterberg–Palladino episode fail to stand out as particularly ‘juicy’; problematic strategies employed by Münsterberg seemed moreover the norm rather than the exception in other examples of ‘boundary-work’ (Gieryn, 1983) not restricted to American history (see, for example, Sommer, in print; Wolffram, 2009; Prince, 1930; Taylor, 1996).^29^ Historical debunking exercises of psychical research have regularly involved intellectual ‘virtues’ that would quickly cost the critic his or her job if employed in the treatment of respectable fields of study. Moreover, far from marking a discrete or closed historical chapter in sociological studies of the rejection of modern parapsychology (the quantitative study of alleged extra-sensory perception and psychokinesis) by psychologists and mainstream scientists have shown that these strategies continue to be employed (see, for example, Collins and Pinch, 1979; Hess, 1993; McClenon, 1984, and Pinch and Collins, 1984).^30^

Apart from the significance of such episodes for academic and scientific core values we usually take for granted (such as intellectual integrity and academic freedom), fostering the taboo of the ‘occult’ has disastrous consequences for historical scholarship. Just to remain with William James studies: whereas the first select compilation of James’ psychical research writings was published in French about two and a half decades after his death (James, 1924), it took almost another four decades for an English compilation to appear (Murphy and Ballou, 1961), and another two and a half for the most recent and comprehensive collection (James, 1986). These ‘apocryphal’ works are obviously necessary additions to the corpus of James studies, but their separate issuing documents the artificial divide that scholars have created between the man’s unorthodox and ‘respectable’ works and achievements, which has enormously complicated a coherent understanding of James. This has been documented, for example, by Marcus Ford (1998), who published an analysis of the James literature up to the late 1990s with regard to the implications of James’ interest and involvement in psychical research. He found that most James scholars have been reluctant even to address James’ active and lasting involvement in psychical research and his conviction of the reality of certain psychic phenomena, let alone discuss the importance of his unorthodox interests for an understanding of his psychological and philosophical writings. Shortly before Ford addressed this problem, Eugene Taylor (1983, 1996) had started to demonstrate the immense significance of James’ involvement in psychical research for his work in psychology and psychopathology, particularly in the period between *The Principles of Psychology* (1890) and *The Varieties of Religious Experience* (1902). ^31^

In fact, prior to authors like Ellenberger and Taylor, professional historians of psychology – the majority of who were and still are trained psychologists – were simply not interested in these issues. Edward Titchener, another contemporary of James vehemently opposing the latter’s advocacy of psychical research, had concluded his assessment of the scientific status of psychical research with a statement that could qualify as a tacit yet powerful epistemological prescription underlying the academic curriculum of modern psychology – and its historiography – up to the present day: ‘No scientifically-minded psychologist believes in telepathy’ (Titchener, 1898: 897). Titchener’s pupil Edwin Boring, the eminent historian of psychology, continued this tradition and even went so far as  retroactively to censor James in a preface to a debunking study by the psychologist Charles E. M. Hansel, whose ‘historical’ part, incidentally, relies on Münsterberg’s Palladino account (Hansel, 1966: 42, 213–14). Selectively quoting from James’ last public statement on psychical research (James, 1909a), where James admitted his inability to account for psychic phenomena with a specific theory, Boring sweepingly disqualified any belief in psychic phenomena by reference to the ubiquitous ‘need to believe’ theory. Surprisingly, he then also praised as exemplary ‘James’s own suspended judgement on psychic research’ (Boring, 1966: xvii), neglecting to mention James’ emphatic statements to the contrary in the same article and elsewhere as far as the very facts in question are concerned.^32^

While pre-1990s James scholarship is perhaps the most conspicuous example of what might be called passive or boundary-work *pace* the historiography of psychology, the visible interest and involvement of other renowned psychologists in the study of psychic phenomena following James (William McDougall, Alfred von Winterstein, J. C. Flügel, Cyril Burt, Constance Long, Gardner Murphy, Hans Eysenck and others) and psychotherapists discussing the occurrence and significance of psychic phenomena in the therapeutic setting (e.g. S. Freud, S. Ferenczy, N. Fodor, J. Ehrenwald, J. Eisenbud, E. Servadio) has also largely failed to be appropriately reflected by historians.^33^ These examples clearly show that interest in alleged psychic phenomena has never been limited to an eccentric or let alone intellectually inferior minority in the psychological community. This also suggest that the unloved sibling of modern psychology has been dissociated from its history mainly by editorial fiat. 

The very vehemence and affectivity of attacks, and the thinly veiled academic contempt towards ‘the Other’ of modern psychology, suggest that what was (and apparently still is) at stake is more than merely intellectual disagreements or problems of professionalization. The traditional historiography of psychical research, dominated by the ‘winners’ of the race for ‘the science of the soul’, reveals fascinating epistemological incommensurabilities and a complex set of interplays between scientific and metaphysical presuppositions in the making and keeping alive of the scientific status of psychology. Thus, revised histories of psychical research and its relationship to psychology with a critical thrust not limited to that which has been viewed with suspicion anyway, offer both a challenge and a promise to historians, the discussion of which the present article hopes to stimulate.^34^

## Notes

I am grateful to Carlos Alvarado, Sonu Shamdasani, Hasok Chang, Liz Valentine and the anonymous reviewers for thoughtful feedback on the original manuscript. My work is funded by a Wellcome Trust History of Medicine PhD studentship.

1. Josiah Royce, undated note in the James–Royce correspondence at Houghton Library, Harvard, cited in Bjork (1983: 67).

2. For a collection of James’ psychical research writings, see James (1986). While James was certainly the most distinguished early US psychologist advocating psychical research, perhaps the most enthusiastic was Harlow Gale of the psychological laboratory at the University of Minnesota (Moore, 1977: 155–6).

3. Cattell, to whom James wrote in 1899 to express gratitude for publishing his replies in *Science* (Skrupskelis and Berkeley, 1992–2004: X, 3), defended James in 1903, when James’ election as a member of the National Academy of Sciences was opposed because of James’ acceptance of the reality of psychic phenomena. Cattell countered that the academy included Christian members embracing the Immaculate Conception and the Resurrection as facts, and that, as ‘the Academy would not reject the greatest psychologist in the world on the ground that he was a Methodist or a Catholic … William James should not be rejected no matter what his views on telepathy and spiritualism’ (cited in Sokal, 2010: 33).

4. Compare, for example, the opposing views regarding alleged psychic phenomena in two leading psychical researchers, Charles Richet and Frederic Myers. Richet, viewing psychic phenomena as challenging scientific anomalies, strictly adhered to scientific materialism and held that supernormal phenomena would eventually become comprehensible in terms of a physiological theory (Wolf, 1993). The research of Myers, on the other hand, was driven by a personal need to find empirical evidence for the spiritual nature of mind and its survival of death, though this did not lead Richet, James, Flournoy and other eminent contemporaries of Myers to dismiss his research as intrinsically flawed (Hamilton, 2009).

5. Though there were overlaps, ‘mental’ mediums produced automatic writing and speech, or conveyed quasi-sensory impressions, while ‘physical’ mediums allegedly produced physical effects such as levitations, telekinesis, materializations, apports, etc. For a concise history of the Palladino controversy, see Alvarado (1993).

6. The popular and scholarly literature often referred to Palladino using her forename only.

7. See, for example, the typology of mediumistic fraud in Eusapia by the psychologist and philosopher Julian Ochorowicz (1896). More than three decades later, Eugen Bleuler defended the work of Albert von Schrenck-Notzing in physical mediumship against accusations to cover fraud (Bleuler, 1930). Bleuler stressed the importance of first-hand experience in psychopathology in order to understand and experimentally control for mediumistic trickery, which he and others considered part and parcel of genuine mediumship.

8. However, James occasionally investigated other physical mediums. See, for example, James (1909b).

9. Emphases are always James’ own.

10. In a letter to Hugo Münsterberg on 17 November 1909, Carrington wrote: ‘In asking E. P. to this country, I did not expect to make one cent from her visit: on the contrary, I expected it would cost me a good deal (as it has done) to bring her here. We have not yet covered expenses!’ Hugo Münsterberg papers, Boston Public Library.

11. Joseph Jastrow also conducted a sitting with Palladino and reported her trickery in popular magazines (1910a, 1910b). Münsterberg’s article, or rather its summary in the *New York Times*, has received more attention than Jastrow’s equally programmatic and problematic reports. Pre-war *NYT* editorials dealing with psychology had a strong focus on Hugo Münsterberg’s rather than any other US psychologist’s work (Dennis, 2002). For details and a criticism of the Jastrow sitting, see Carrington (1954: 244–7).

12. For biographical accounts, see, for instance, Hale (1980) and Spillmann and Spillmann (1993).

13. This did not prevent him from publishing in the proceedings of the Gesellschaft für psychologische Forschung (Münsterberg, 1893), which was an amalgamate of the leading psychical research societies in Berlin and Munich. On the German psychological/psychical research societies see Kurzweg (1976) and Wolffram (2009).

14. However, as Hale (1980) argued, Münsterberg’s conversion from Judaism to Christianity was likely motivated by political rather than religious considerations.

15. Münsterberg was also living in Cambridge at the time, in close vicinity to the James family. Regarding Royce’s refusal to test Leonora Piper, James wrote on 19 May 1894 to his wife Alice that he had lost his temper with him (Skrupskelis and Berkeley, 1992 –2004: VII, 611). Titchener continued to refuse investigating Leonora Piper after James’ letter.

16. See James to Schiller, 19 October 1899 and 17 November 1899, Skrupskelis and Berkeley (1992–2004: IX, 63, 86).

17. Later, Münsterberg ‘defended’ James in the *New York Evening Post* (8 January 1901) as well as in the *Nation* (17 January 1901) against a newspaper article claiming that James had lost his mind, which the reporter responsible for the statement thought was indicated by James’ endorsing a spiritualist medium (Leonora Piper). Münsterberg, writing in his capacity as chairman of the Harvard Department of Philosophy, set the record straight but also used the opportunity to explicitly posit himself as the main critic of James’ involvement in psychical research. James was not overly happy about Münsterberg’s ‘defence’. On 1 February 1901 he wrote to George Herbert Palmer: ‘I wish he hadn’t been quite so lengthy in his argumentative vindication of my sanity in the E[vening] P[ost], which to my profound horror I find reproduced in the more permanent pp. of the Nation!’ (Skrupskelis and Berkeley, 1992–2004: IX, 417).

18. Palladino scrapbook in the archives of the Society for Psychical Research, Cambridge University Library, SPR.MS44/4/2/70. Eusapia’s surname has been spelt inconsistently in the literature, Italian authors tending to ‘Paladino’ and English to ‘Palladino’.

19. James refers to a report of Palladino experiments with Henri Bergson and Marie and Pierre Curie as co-investigators (Courtier, 1908).

20. James to Carrington, 15 June 1909, published in Carrington (1957: 41–2). A copy of this letter is in the James papers, Houghton Library, Harvard (MS Am 1092.1.[20]).

21. Paul Dennis (2002) found that Münsterberg was the one US psychologist receiving the most extensive coverage in editorials and other articles in the *New York Times* between 1904 and 1914.

22. The original article contains the following sentence: ‘It was a scream as if a dagger had stabbed Eusapia right through the heart’ (Münsterberg, 1910: 571).

23. In his article, Münsterberg had justified his refusal to consider supernormal phenomena as even theoretically possible by stating that he was ‘no pragmatist’ (Münsterberg, 1912: 147).

24. According to Eric J. Dingwall (1962: 211), the foot-grabber was Edgar T. Scott. For information corroborating James’ accusation and casting doubt on further crucial details of Münsterberg’s report, see Carrington (1910; 1913: 127–215; 1954: 107– 17). See also Flournoy’s critique of Münsterberg’s article (Flournoy, 1911: 282–8).

25. Münsterberg papers, Boston Public Library.

26. Carrington refers to his letter to Münsterberg, 31 January 1910, Münsterberg papers, Boston Public Library, in which Carrington protested against the treatment of Eusapia and himself by Münsterberg, accusing him of wilful deception and requesting compensation by paying the fee which he had waived for Münsterberg.

27. Sudden screams, outbursts of weeping and laughter, loud moans, coughs, hiccups, yawning and other noises were no unusual incidents in Palladino’s sittings.

28. The addendum ‘as it has been managed’ indicates disagreements between Hyslop and Carrington beyond matters concerning the handling of the American Palladino sittings.

29. Ferdinand Schiller in England, for example, claimed that his confidence in the intellectual integrity of professional psychologists had been shaken as a result of … the unfortunate outcome of my only attempt to enlist an experimental psychologist’s co-operation in a ‘psychical’ experiment’. He took advantage of the opportunity to secure the failure of the experiment. No doubt his scientific conscience permitted, nay, persuaded, him to protect ‘science’ against the possible inroads of ‘superstition’ by such means, but after this I naturally incline to guard myself against the possibilities of deception on *both* sides. For it is decidedly humiliating to have escaped the wiles of the professional mediums only to fall a victim to the excessive zeal of a professorial psychologist, whose good faith one had taken for granted! (Schiller, 1899: 359; original emphases)

30. This is of course not to say that psychical research and modern parapsychology stand out as the only disciplines that have received unfair treatment (see, for example, Mauskopf, 1979; Wallis, 1979). The case of psychical research, unlike other stereotypical ‘pseudo-sciences’ such as astrology, alchemy, phrenology, UFOlogy, crypto-zoology and cold fusion research, derives its particular interest not only from its historical significance as a hitherto largely overlooked ‘force’ in the making of modern psychology; unlike these other disputed fields modern parapsychology is (apart, perhaps, from alternative medicine) the only – or at least most visible – one that still attempts to gain access into academia. For example, while university-based departments of parapsychology are globally on the decline, the Parapsychological Association (an umbrella organization of parapsychological researchers) is still a member of the AAAS.

31. The recent Harvard-based online presence in celebration of the centenary of James’ death breaks with the ‘psychical taboo’ tradition and includes a neutrally phrased section on James and psychical research. See: http://hcl.harvard.edu/libraries/houghton/exhibits/james

32. James is unambiguous when he writes: ‘As to there being such real natural types of phenomena ignored by orthodox science, I am not baffled at all, for I am fully convinced of it’ (1909a: 587).

33. An obvious exception has been the Jung scholarship (see, for example, Shamdasani, 2003).

34. One of the anonymous reviewers raised the concern that my claim regarding the contributions by psychical researchers to fledgling psychology (see Introduction) was lacking in originality, since it was believed this constituted common ‘working knowledge’ among current historians of psychology. This may well be the case; however, to my knowledge no historian of psychology has ever provided a summary of these contributions or delivered a concise positive claim to this effect *in print*, or revealed and problematized in detail methods commonly employed to marginalize certain kinds of epistemic deviance. These lacunae are at least compatible with – if not in support of – my broader thesis of historiography as passive boundary-work. In a similar vein, the same reviewer was also concerned that I overstate the case by inflating the apparently extreme example of Münsterberg as representative of the kind of boundary-work exercised by psychologists, and the belief was expressed that such behaviour would surely be sanctioned nowadays. Both the historical and sociological literature which I cite in support of my claims to the contrary clearly shows otherwise. Moreover, and as mentioned in the Introduction, to make a stronger case this article had originally intended to include and discuss the historical relevance of a similar episode, G. Stanley Hall’s and Amy Tanner’s staged ‘exposure’ of Leonora Piper (which even involved physical abuse) shortly after James’ death (Tanner, 1910), as well as its overall positive reception in the psychological press, which space limitations forced me to reserve for a separate occasion. Finally, regarding ongoing boundary-work by psychologists, complaints very similar to those which James, Schiller, Flournoy, Sidgwick, W. F. Prince and other psychical researchers had levelled against Münsterberg, Hall, Jastrow, Titchener, Cattell, etc., continue to be made against certain present-day professional debunkers and self-styled media experts of science and parapsychology, many of whom are psychologists, and whose work is widely cited as authoritative and supported by other psychologists and scientists. The relevant sociology of science literature from the late 1970s, which I partially cite, demonstrates a clear continuity of ethically problematic boundary-work, and more recent works suggest that very little has changed in the last few decades (see, for example, Carter, 2007, 2010; McLuhan, 2010). Even though these accusations of intellectual dishonesty continue to be serious, appear well-substantiated and have in essence remained unanswered by the targets of criticism, their reception is limited to circles outside the scientific and psychological establishment, and they continuously fail to be acknowledged or discussed in the mainstream science and psychology literature.
